# A Population Rate Code of Auditory Space in the Human Cortex

**DOI:** 10.1371/journal.pone.0007600

**Published:** 2009-10-26

**Authors:** Nelli H. Salminen, Patrick J. C. May, Paavo Alku, Hannu Tiitinen

**Affiliations:** 1 Department of Biomedical Engineering and Computational Science, Helsinki University of Technology, Helsinki, Finland; 2 Department of Signal Processing and Acoustics, Helsinki University of Technology, Helsinki, Finland; 3 BioMag Laboratory, Hospital District of Helsinki and Uusimaa HUSLAB, Helsinki University Central Hospital, Helsinki, Finland; Ludwig Maximilians University Munich, Germany

## Abstract

**Background:**

Previous work on the human auditory cortex has revealed areas specialized in spatial processing but how the neurons in these areas represent the location of a sound source remains unknown.

**Methodology/Principal Findings:**

Here, we performed a magnetoencephalography (MEG) experiment with the aim of revealing the neural code of auditory space implemented by the human cortex. In a stimulus-specific adaptation paradigm, realistic spatial sound stimuli were presented in pairs of adaptor and probe locations. We found that the attenuation of the N1m response depended strongly on the spatial arrangement of the two sound sources. These location-specific effects showed that sounds originating from locations within the same hemifield activated the same neuronal population regardless of the spatial separation between the sound sources. In contrast, sounds originating from opposite hemifields activated separate groups of neurons.

**Conclusions/Significance:**

These results are highly consistent with a rate code of spatial location formed by two opponent populations, one tuned to locations in the left and the other to those in the right. This indicates that the neuronal code of sound source location implemented by the human auditory cortex is similar to that previously found in other primates.

## Introduction

Auditory localization poses a unique challenge to the nervous system. In vision and touch the sensory receptors represent space in a topographic manner and, thus, location information is already available in the organization of the neuronal periphery. However, the auditory system needs to determine source locations from sensors organized according to sound frequency. Therefore, forming a neuronal representation of auditory space requires computations where localization cues are extracted and combined over the whole spectrum of the sound. In the human auditory cortex, certain areas seem to be specialized in performing these computations [1]–[3]. When sound stimuli are presented from several locations instead of only one the activity in the posterior regions increases [1], [4], [5]. While this suggests the existence of spatially selective neurons in the posterior auditory cortex, how these neurons represent auditory space remains unknown.

Although the auditory periphery is not organized according to spatial location, a topographical place code consistent with the spatial representations of stimulus features in other modalities could be reached through neuronal computations. This was first suggested in the delay line model by Jeffress [6] and, later, other computational mechanisms to achieve neuronal selectivity for sound source location have been described [7]. The place code of auditory space has received its strongest support from studies focused on the encoding of the interaural time difference (ITD) which is the dominant cue for sound source localization in low frequencies. ITD tuning consistent with a place code has been found in single neurons of the mammalian superior olive [8]–[10], inferior colliculus [11]–[14], superior colliculus [15]–[17], and medial geniculate body of the thalamus [18], [19], as well as in the owl auditory nuclei [20]–[22]. Further, a topographical place code of spatial location has been observed in the mammalian superior colliculus [15]–[17], and in the owl auditory nuclei [20], [21] utilizing sounds presented from loudspeakers and thus including all localization cues. In these maps, the representation of space is often non-uniform. Frontal locations are encoded by a larger number of neurons and the receptive fields are narrower than for rear locations [12], [20], [21]. This has been interpreted as a neuronal substrate of better behavioral localization of sound sources in front than of those in the rear.

Alternatively, auditory space could be represented by a population rate code of two opponent populations: one preferentially activated by sound sources to the left and the other by those to the right of the perceiver. The location of a sound source would be then encoded in the relative level of activity in these two groups of neurons. The opponent populations were originally proposed as a model of the integration of localization cues studied in psychophysical research [23], [24] and was later formulated as a physiological model [25]. Spatial selectivity consistent with the population rate code has been found in several neurophysiological studies utilizing ITD, interaural level difference (ILD) or fully realistic spatial sound containing both of these cues. Neurons with large spatial receptive fields centered at lateral locations have been encountered in the mammalian superior olive [26]–[28], inferior colliculus [29]–[33], medial geniculate body [34], [35], and auditory cortex [36]–[39]. In subcortical structures, spatially selective neurons are typically activated only by sound sources in the hemifield opposite to the nucleus [29]–[31]. In the auditory cortices, neurons tuned to both hemifields have been found although those tuned to the contralateral hemifield are still in majority [37]–[39]. In the monkey auditory cortex, the majority of spatially selective neurons are tuned to lateral locations [36], [38], [39], making the population rate code a strong candidate for the auditory spatial representation in the human cortex. The rate code is, however, limited to encoding locations only in left-right dimension: it cannot describe the neuronal mechanisms that allow front-back discrimination or the perception of sound source elevation.

**Figure 1:**
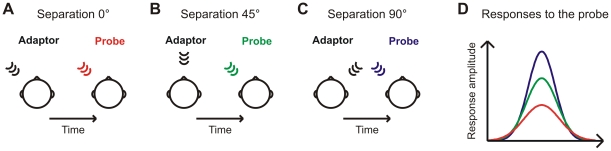
Illustration of stimulus-specific adaptation. Sounds are presented sequentially from two locations: an adaptor and a probe location. (A) When the two sound sources are at the same location they activate the same neuronal population. The attenuation caused by the adaptor is maximal and, consequently, the response measured to the probe is small (D, red). (B) A spatial separation is introduced between the sound sources. Assuming that there is selectivity for sound source location, some of the neurons that are responsive to the probe location are not activated by the adaptor. These neurons remain unaffected by the adaptor and contribute to a less attenuated response to the probe (D, green). (C) When the separation between the sound sources is further increased the number of neurons responsive to the probe but not to the adaptor also grows. Accordingly, the response to the probe becomes stronger (D, blue).

Studying the single-neuron selectivity to sound source location in the human brain is problematic as the non-invasive methods available fuse the activity of large neuronal populations into aggregate signals. Thus, revealing the auditory spatial code requires a method capable of measuring the spatial selectivity of neurons even when their activity is represented in a spatially summed signal. For this purpose, a previous study successfully used the N1 response, a prominent deflection in the event-related potential (ERP) peaking at around 100 ms after sound onset [40]. In a stimulus-specific adaptation paradigm, two alternating sounds, an adaptor and a probe, were sequentially presented and the attenuation caused by the adaptor on the N1 response to the probe was measured (Fig. 1). When the two sounds were presented from the same location, the attenuation was maximal. However, as a spatial separation was introduced between the adaptor and the probe the N1 response to the probe increased as a function of the separation between the two sound source locations. These findings were interpreted to arise from a population of spatially selective neurons. Specifically, the attenuation of the N1 reflects the degree to which the respective set of neurons selectively responding to the probe and the adaptor location overlap. When the probe and the adaptor are at the same location they activate the same neurons and this leads to maximal attenuation. However, when the adaptor and the probe are at different locations, some of the spatially selective neurons are activated by the probe but not by the adaptor. These neurons are left outside the attenuating influence of the adaptor and, consequently, give rise to a larger N1 response. These results on N1 attenuation therefore demonstrate that neurons in the human auditory cortex are spatially selective. Unfortunately, due to the use of only a limited set of source locations they do not reveal whether the spatial receptive fields of these neurons correspond to the place code or the rate code.

**Figure 2:**
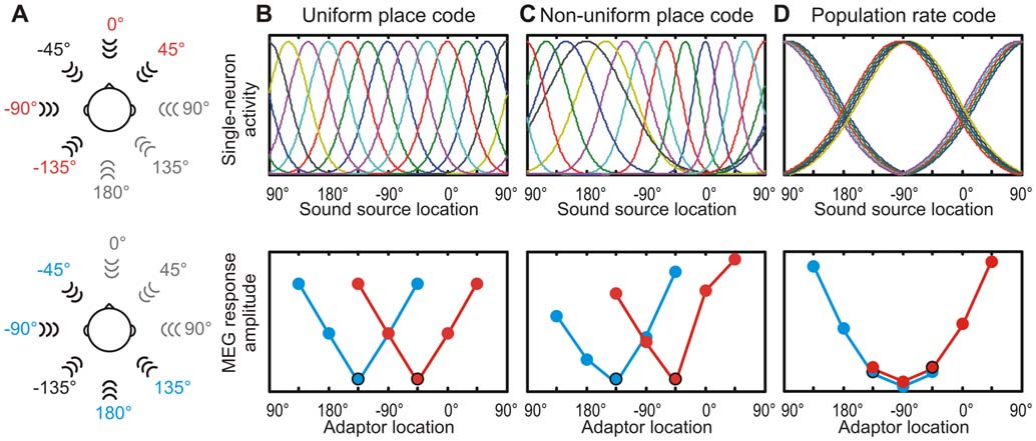
Illustration of the experimental setup and the hypotheses. (A) Stimulus specific-adaptation was measured for a probe located in the left frontal hemifield at −45°. The probe was coupled with adaptors presented from five locations (red). A similar setup was constructed for a probe in the left rear hemifield at −135° (blue). (B) In the place code, auditory space is uniformly represented by auditory cortical neurons without any location-dependent variation in the receptive fields (only a subset of the curves is plotted). The uniform place code predicts that the response amplitude to the probe depends solely on the separation between the probe and the adaptor location. (C) In a variation of the place code, the receptive fields are narrowest for neurons tuned to front and broadest for those tuned to rear locations. Consequently, the least attenuated responses are found for frontal adaptor locations and strongest adaptation occurs for rear locations. The laterally presented adaptors lead to intermediate responses. (D) In the population rate code, neurons are maximally activated by laterally presented sounds and have wide spatial tuning curves. Each cortical hemisphere contains both neurons tuned to the left hemifield and those tuned to the right hemifield. When the adaptor and probe are presented in opposite hemifields, these activate different neuronal populations and the response to the probe is of large amplitude. In contrast, adaptors in the same hemifield as the probe activate the same population and, thus, responses are attenuated.

In the present study, we measured the stimulus-specific adaptation effects for a wide set of sound source locations with the aim of revealing whether human cortex utilizes the place code or the rate code for representing auditory space. To this end, human subjects were presented with realistic spatial sound stimuli that included the ITD and ILD, as well as the spectral cues arising out of the modulations due to the shape of the head, the pinna, and the body. Three experimental predictions were formulated based on a uniform and a non-uniform place code and a population rate code. The uniform place code predicts that the N1 amplitude increases as the separation between the probe and the adaptor grows (Fig. 2B) and that this increase is independent of the absolute location of the two sound sources. For the non-uniform place code, the increase of the N1 amplitude as a function of the separation between the probe and the adaptor depends on the width of spatial tuning (Fig. 2C). Narrow tuning for frontal locations, where behavioral localization is best, leads to a large increase in the N1 response already for small stimulus separations. Wide spatial tuning for rear locations requires a much larger stimulus separation to produce an equivalent increase in the N1 amplitude. Finally, the rate code predicts that the N1 response of each cortical hemisphere reflects the compound activity of one population tuned to the left hemifield and another tuned to the right hemifield. Thus, the N1 amplitude is largely determined by whether the probe and the adaptor are in the same hemifield or in the opposite ones (Fig. 2D). When the two sound sources are in the same hemifield they activate the same neuronal population and the N1 responses are of low amplitude. When the probe and the adaptor are in opposite hemifields they activate different populations and, consequently, N1 responses are large in amplitude. To test these predictions we conducted a magnetoencephalography (MEG) experiment on human subjects. Realistic spatial sound stimuli were prepared individually for each subject and presented in varying probe-adaptor pairs, and the stimulus-specific adaptation of the N1m, the magnetic counterpart of the N1 response, was measured.

## Results

**Figure 3:**
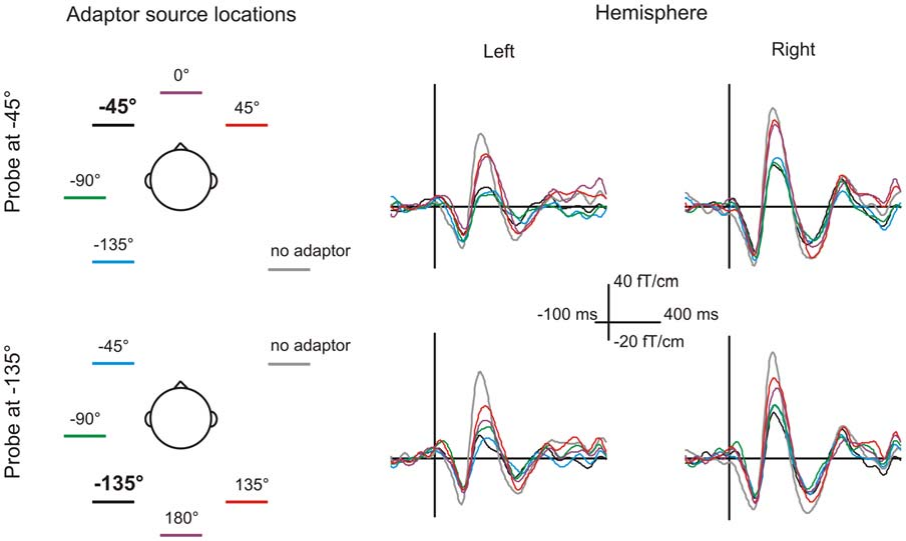
Grand-averaged event-related fields measured from the left and right hemisphere. The smallest responses, that is, strongest adaptation was found for the conditions in which the adaptor and the probe were at the same location (black) or when the adaptor was in the same hemifield (blue and green). For adaptors at the midline or in the right hemifield (purple and red) the responses were larger and, thus, adaptation was weaker. Largest responses were found when no adaptor was presented (gray).

**Figure 4:**
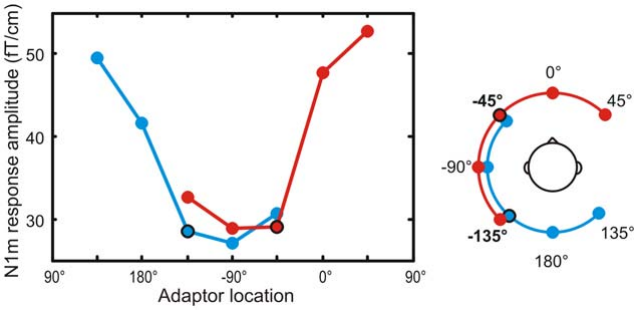
The average amplitude of the right-hemispheric N1m response to the frontal and rear probes. The responses were prominent when adaptors were located in front, in the rear, or in the right hemifield. When the adaptors were presented in the same (left) hemifield as the probe, response amplitudes were small. This is consistent with auditory cortical neurons having laterally centered and wide spatial tuning (for comparison, see Fig. 2D).

The amplitude of the N1m response was measured for a probe sound at −45° presented in the context of five different adaptor locations (Fig. 2A). The amplitude depended strongly on the adaptor location (F[5], [55] = 480, p<0.001). As could be expected based on previous research [40], the amplitude of the right-hemispheric N1m to the probe grew as a function of the separation between the probe and the adaptor location. When the adaptors were located clockwise towards the right hemifield (Fig. 3, top, Fig. 4, red), the N1m responses increased from 30.0 fT/cm for the adaptor at −45° to 47.7 fT/cm for the adaptor at 0° and, finally, to 54.9 fT/cm for the adaptor at 45° (p<0.05 for all comparisons). The latter was close to the amplitude of 61.6 fT/cm found in the no-adaptor condition. Thus, the further away the adaptor was from the probe, the weaker its attenuating effect on the N1m became. In contrast, when the adaptors were at lateral locations in the left hemifield, the N1m amplitude was independent of the separation between the probe and the adaptor. The amplitudes were 30.0, 29.0, and 32.7 fT/cm for adaptors at −45°, −90°, and −135°, respectively (p = n.s.). Thus, when the probe and the adaptor were within the same hemifield, all adaptors were equally effective regardless of the spatial separation.

To compare the spatial tuning properties for front and rear space, the N1m responses were also measured for a probe at −135° (Fig. 2A). These results were highly similar to those obtained with the probe at −45° (F[1], [11] = 1.8, p = n.s.). When the adaptors were located counter-clockwise towards the right hemifield from the probe, the responses grew as a function of separation between the probe and the adaptor. With adaptors at −135°, 180°, or 135° (Fig. 3, bottom, Fig. 4, blue) the respective right-hemispheric N1m response amplitudes were 28.5 fT/cm, 41.6 fT/cm, 49.5 fT/cm (p<0.05). For adaptor locations within the left hemifield at −135°, −90°, and −45° the respective N1m amplitudes were 28.5, 27.1, and 30.6 fT/cm (p = n.s.). Thus, the adaptors were, again, all equally effective if they were in the left hemifield but when they crossed the midline to the right hemifield the strength of adaptation depended on the separation between the probe and the adaptor location.

Although the experimental setup was specifically designed to engage the right-hemispheric auditory areas, the amplitude of the left-hemispheric N1m also showed significant variation depending on the adaptor condition (Fig. 3). The left-hemispheric N1m responses followed the same pattern of amplitude variation as the right-hemispheric ones. They were larger when the adaptors were at the midline or in the right hemifield than when the adaptors were in the left hemifield (p<0.05). However, the left-hemispheric responses were, on the average, half the magnitude of the right-hemispheric ones (22.2 & 41.1 fT/cm, for the left and right hemisphere, respectively, F[1], [11] = 19.6, p<0.01) and, correspondingly, the location-dependent variation of the amplitude of the N1m response was smaller in the left than in the right hemisphere. This is consistent with a smaller left- than right-hemispheric population of neurons responding to the probe presented in the left hemifield.

**Figure 5:**
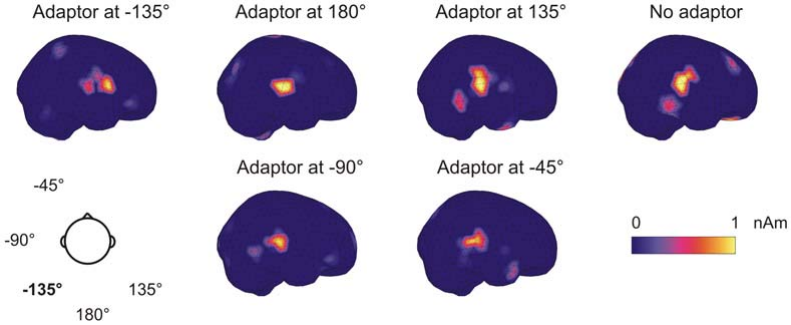
Minimum current estimates of a representative subject obtained at the N1m peak latency. In all conditions, the activity originated mainly from the temporal areas in the proximity of auditory cortex.

Minimum current estimates (MCE) confirmed that the activity occurring during the N1m response took place in the temporal areas of the cortex (Fig. 5). The response amplitudes derived from the MCE analyses were consistent with the location-specific effect found in the previous analyses (F[5], [55] = 33.3, p<0.001). Maximal responses were measured for the no-adaptor condition (16.7 & 10.7 nAm, for right and left hemisphere, respectively) and minimal responses for adaptor locations within the left hemifield (5.7–6.4 & 4.0–4.9 nAm). When the adaptors were at the midline or in the right hemifield, responses were of intermediate amplitudes (10.0–11.3 & 5.9–7.0 nAm).

The latencies of the N1m responses varied according to the adaptor condition (F[5], [55] = 4.9, p<0.001) and no differences were found between the two hemispheres (F[1], [11] = 3.0, p = n.s.). The shortest latencies occurred, on the average, at 104 ms for the conditions where sounds were presented only from the probe location, that is, in the no-adaptor condition or when the adaptor was presented from the same location as the probe. The longest latencies, at 110 ms, were measured when the adaptors were at the midline or in the right hemifield (locations 0°, 45°, 180°, and 135°, p<0.05). The response latencies in the conditions with adaptors within the left hemifield (at −45°, −90°, and −135°) fell between these two values, at 107 ms, but their difference from the other conditions was not significant (p = n.s.).

## Discussion

The purpose of the present study was to reveal the encoding strategy used by the human cortical neurons to represent realistic spatial sound containing all localization cues. With an experimental paradigm based on the stimulus-specific adaptation of the N1m response we were able to describe the previously unknown spatial tuning properties of neurons in the human auditory cortex. We found strong location-specific effects in the attenuation caused by an adaptor on the N1m response to a probe sound. When the adaptor was in the same hemifield as the probe, response amplitudes were low and independent of the spatial separation between the two sources. In contrast, when the adaptor was at the midline or in the opposite hemifield, responses to the probe were prominent and approached in amplitude those measured without any adaptor. These findings correspond best to the experimental predictions based on a population rate code of auditory space (see Figs. 2D & 4). Thus, the present study indicates that the human auditory cortex represents sound source location with two populations of spatially sensitive neurons, one preferring sound sources to the left and the other to the right of the perceiver.

The current results are corroborated by those of previous studies [3], [40] where a spatial separation between two sound sources in the front led to increased N1 response amplitudes. These studies, however, utilized only one probe location and a limited set of adaptor locations in front of the subject. For these frontal sound source locations, the place code and the rate code predict similar adaptation of the N1 response (see Fig. 2B–D). Therefore, previous studies do not allow conclusions on the shape of the underlying spatial receptive fields. Here, by measuring the location-specific adaptation on the N1m response for a wide set of direction angles we were able establish that the representation of realistic spatial sound in the human auditory cortex is based on a rate code.

**Figure 6:**
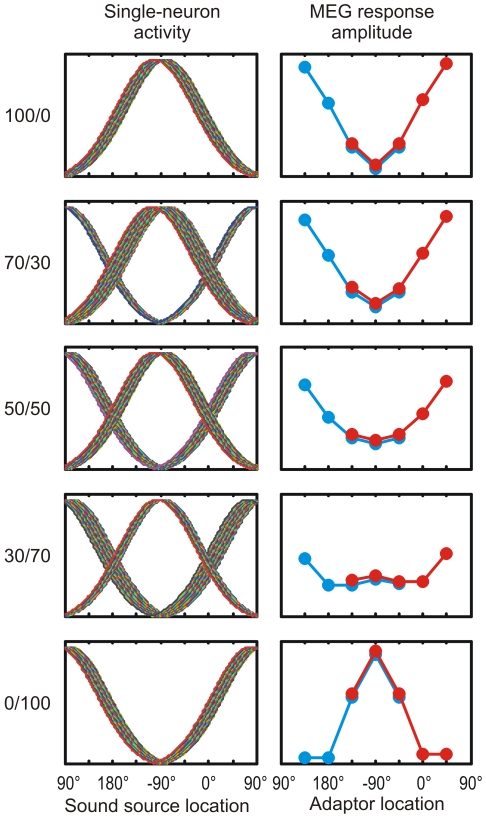
Experimental predictions of the population rate code derived for different numbers of neurons tuned to the left and right hemifields. When the proportion of neurons tuned to the left exceeded 30% of all neurons, the predicted MEG results resembled those obtained in the present experiment.

The spatial sound stimuli contained, among all other localization cues, an interaural level difference. As the auditory pathways cross and each cortical hemisphere receives more input from the contralateral ear, mechanisms unrelated to sound source localization might contribute to the variation of the amplitude of the N1m response. Our results are, however, not consistent with a significant contribution from such mechanisms. For example, the N1m amplitude could reflect simple effects of sound level and crossing neural pathways, in which case we would expect to see opposite patterns of variation in the response amplitudes of the two cortical hemispheres. This was, however, not the case: the adaptors to the left caused strong attenuation and those to the right weak attenuation in both cortical hemispheres. This similarity between the left- and right-hemispheric results probably reflects a similarity between the spatially selective neurons giving rise to them. As the probe sound was always to the left of the subject, the responses of both hemispheres presumably reflect the activity of the neuronal population tuned to the left hemifield. This population is possibly larger in the right than in the left hemisphere as larger response amplitudes were measured from the right than from the left. Thus, our results are consistent with a population rate code where each cortical hemisphere comprises both left-tuned and right-tuned populations of neurons (Fig. 6), with the contralaterally tuned population possibly being larger than the ipsilaterally tuned one. Such a distribution of tuning properties is in line with intracortical recordings [37], [38].

Our results probably reflect the compound activity of several types of cortical neurons. These could include, at least, binaural neurons sensitive to interaural difference in time and level (ITD and ILD, respectively) as well as monaural neurons whose activity reflects increases in monaural sound level. The contribution of the monaural neurons was, however, unlikely to be significant as the differences in sound level they experienced were relatively small, 5 dB or less, while the corresponding increases in the amplitude of the N1m response were nearly two-fold. Therefore, our results are likely to reflect mainly the activity of binaural neurons. Single-unit [41]–[43] and human brain imaging data [44]–[47] show that neurons in the auditory cortex are sensitive to manipulations of both ITD and ILD cues alone. As both of these spatial cues were included in our stimuli, their contributions cannot be disentangled in the present findings. The strongest support for the place code of auditory space arises from studies focused on ITD [9], [10] while studies including ILD as the only cue or as part of free-field stimuli are consistent with the population rate code [26], [27], [30], [33], [38], [39]. Thus, the possibility remains that the spatial cues are processed in different ways in the auditory cortex and that our findings predominantly reflect the representation of ILD. This is an important question for future experimental work to address.

Posterior auditory areas seem to have a special role in spatial processing both in humans and in monkeys. Although spatial selectivity is found in both anterior and posterior belt areas of the monkey auditory cortex, the number of spatially selective neurons is greater and the spatial receptive fields are more resistant to variations in sound level in the posterior areas [38], [48], [49]. Similarly, greater spatial selectivity has been found in the posterior than in the anterior areas of the human auditory cortex [3]. Furthermore, the planum temporale in the human posterior auditory cortex shows increased activity to the presentation of moving sound sources compared to a stationary sound source [1] or to stationary sounds from multiple locations presented either sequentially [4], [5] or concurrently [2]. Given that the planum temporale is a major contributor to the N1m response [50], [51], it is likely that the selectivity found here reflects the specialization of posterior areas to spatial processing.

Our non-invasive findings on the human auditory cortex are consistent with the wide, laterally centered spatial receptive fields found in several invasive neurophysiological studies of the auditory cortices of animal models indicating the presence of a rate code [36]–[39]. Laterally centered receptive fields form the majority of spatially selective neurons in the monkey auditory cortex [36], [38], [39] and they are found in all auditory cortical fields studied in the cat [52] and in the monkey [38]. In mammals, indications of the rate code are found also in the superior olive [26]–[28], the inferior colliculus [29]–[33], and in medial geniculate body of the thalamus [34], [35]. However, in the mammalian superior olive and inferior and superior colliculi and in the owl auditory system, results consistent with the place code have been reported [8]–[17], [20]–[22]. These inconsistencies could arise from differences between the various species studied. The owl, for instance, is unique in terms of the acoustical cues produced by the shape of the ears and in how these cues are utilized in behavioral sound source localization. Consequently, the computational strategies of the owl brain in spatial processing might not be comparable to those utilized by the mammals [53], [54]. Furthermore, in the mammalian species, the size of the head determines the range of naturally occurring interaural time delays and the frequency range at which the head shadows the sound signal leading to an interaural intensity difference. Other factors influencing the acoustical information useful for sound source localization are the hearing range of the animal and the shape of the pinnae. Whether a place code or a rate code is the better strategy for extracting and representing auditory spatial information could depend on these species-specific features in the spatial cues [55].

At first glance, the population rate code may seem to be at odds with behavioral performance on sound source localization. In the rate code the neuronal resources are dedicated to encoding the far left and right while human localization behavior suggests that the representation is densest for frontal locations. This apparent discrepancy can, however, be resolved by considering that in the rate code each neuron contributes to the representation of all sounds, not just those eliciting maximal activity [30]–[32], [56], [57]. In these neurons, the level of activity changes very little in response to small changes in sound location to the far left or far right. In frontal directions, the same change, however, leads to a much larger change in the pattern of neuronal activity and, thus, to better discriminability between sound sources close to the midline than between those at lateral locations [57]. The population rate code is, however, limited to accounting only for sound source lateralization in the horizontal plane. Below and above the horizontal plane, sound source localization relies largely on spectral cues produced by the filtering effects of the pinnae and the head [58]. These cues are used in sound localization very effectively but the brain processes related to them are poorly understood. For these purposes, other spatial codes than those tested here may exist, such as other shapes of spatial receptive fields [59], [60] or neural codes based on spike timing [61]–[63]. Thus, an important challenge for future research is to extend the theories and experimental work to deal with the full three dimensions of the auditory space.

While there is no straightforward link between single-cell activity and non-invasive measurements, the stimulus-specific adaptation paradigm appears to offer an effective way to interpret MEG results in terms of single-neuron receptive fields. The paradigm capitalizes on the location-specific adaptation of spatially selective neurons to make the shape and size of the receptive fields visible even in the large-scale brain responses. The stimulus-specific adaptation of the N1m response could be expanded to the study of the processing of various other sound features such as sound frequency or intensity, the identity of environmental sound, or speech sounds. This could provide an interesting opportunity for mapping the strategies that human cortex uses to deal with the complex information of the auditory environment.

## Materials and Methods

### Subjects

Fourteen healthy subjects (mean age 25, standard deviation 5 years) participated in the study with written informed consent and with the approval of the Ethical Committee of Helsinki University Central Hospital. All subjects reported having normal hearing and being right-handed. The data of two subjects were discarded due to a poor signal-to-noise ratio. During the experiments, subjects were under instruction to ignore the auditory stimulation and to focus on watching a self-selected silent film.

### Spatial Stimuli

The spatial sound stimuli were individually prepared for each subject. Miniature microphones were placed at the entrance of the ear canals of the subject. Eight loudspeakers were arranged in a circle and spanned the horizontal plane in steps of 45° (Fig. 2A). The loudspeakers were placed at a distance of 1.3 meters from the center of the circle where the subject was seated. The height of the loudspeakers was equal to the vertical distance of the subject's ears from the floor (1.2 meters). A 200-ms white-noise stimulus was sequentially presented from each loudspeaker. The recordings were performed in a slightly reverberant listening room adhering to the ITU-R BS.116 standard (measured reverberation time *T*_60_ = 0.3 s). In the MEG measurement, these recordings were presented binaurally through a custom-made tube phone system whose frequency response was digitally equalized at 100 Hz–10 kHz.

### Experimental Procedure

In the MEG measurements, the stimulus-specific adaptation paradigm was implemented by presenting the spatial sounds in blocks of two alternating sound source directions: the probe location and the adaptor location. The paradigm capitalizes on the adaptation, or masking, that a sound incurs on the responses to subsequent sounds, and which is visible in invasive recordings of the cortex [64]–[66] and in the N1 response [67]. This effect is stimulus-specific so that adaptor sounds with different properties from the probe, for example in terms of sound frequency, are not equally effective as adaptors as an identical sound would be. That is, the strength of the adaptation depends on the extent to which the adaptor sound frequency falls into the frequency receptive field of the neuron [64]. Stimulus-specific adaptation occurs in the cortex over several time scales ranging from tens of milliseconds to several seconds [65], [66].

The onset-to-onset interstimulus interval was 1 s and, thus, a probe sound occurred every 2 s in each block. The sound source directions were chosen to span the auditory space in the front, to the left, and in the rear. Two probe sound source locations were used. These were both 45° from the midline, one at a frontal (−45°) and the other at a rear (−135°) location. The left hemifield was chosen as the location of the sound stimuli to ideally target the right-hemispheric brain areas, which are more responsive to the spatial quality of sound [1], [2], [44], [68]–[71].

Angular separations between the adaptor and the probe of 0°, 45°, and 90° were used in both clockwise and counter-clockwise directions (Fig. 2A). The adaptors were either within the same (left) hemifield as the probe location (at −90° or −135° for the front probe and at −90° and −45° for the rear probe) or towards the opposite (right) hemifield (at 0° and 45° for the front probe and at 180° and 135° for the rear probe). This resulted in five adaptor locations for each probe stimulus, two within the same hemifield, two towards the opposite hemifield, and one at the same location as the probe. A no-adaptor control block with the probe stimulus presented with an ISI of 2 s without intervening adaptors was also included. Altogether, there were eleven blocks whose presentation order was counterbalanced across subjects.

The use of only two fixed probe locations to map the horizontal plane instead of using several probes at varying locations was dictated by the variation of the N1m amplitude as a function of sound source direction [44], [69]–[71]. The N1m is largest for sounds contralateral to the hemisphere from which it is measured and smallest for ipsilaterally presented sounds. The N1m amplitudes for sources in front and to the rear of the subject are intermediate. Thus, to ensure that the variation in the N1m amplitude is due to adaptation effects and not to the location of the probe, it was crucial to make comparisons only between responses to the same sound source location but presented in different contexts.

### MEG Data Acquisition

Brain responses were recorded with a 306-channel whole-head MEG device (Vectorview 4-D, Neuromag Oy, Finland). Data was recorded with a passband of 0.03–200 Hz and a sampling rate of 600 Hz and averaged online from 100 ms before stimulus onset to 400 ms after. A minimum of 150 responses was acquired for each sound source direction and adaptation condition. Horizontal and vertical eye movements were measured with electrodes, and epochs which included absolute deviations larger than 150 µV were automatically discarded. The averaged brain responses were bandpass-filtered at 1–30 Hz and baseline corrected with respect to a 100-ms pre-stimulus period.

For each hemisphere and subject, data from the channel-pair with maximal response amplitudes was chosen for further analysis. The N1m response was quantified from the amplitude of the vector sum obtained from the channel pair as the peak amplitude in the 80–120 ms latency range. To visualize the spatial extent of the cortical activity, and to verify that it originated from the temporal areas, minimum current estimates (MCEs) [72] were obtained from a 20-ms time window centered at the N1m peak latency. The evoked responses were detrended with respect to a 300-ms poststimulus period and lowpass filtered at 30 Hz for the MCE analysis. A realistic head-model (standard-bem, NeuroMag) was used from which spherically shaped regions of interest placed in the left and right temporal lobes were chosen.

### Statistical Analyses

The analyses focused on the responses elicited by the two probe locations in each adaptation condition. Repeated-measures analyses of variance (ANOVAs) were performed for the peak amplitudes and latencies of the N1m response of the two hemispheres. The dependent factors were the hemisphere (right and left), the two probe sound locations (−45° and −135°) and the six adaptor conditions. Newman-Keuls post-hoc comparisons were performed when appropriate.

### Formulation of Experimental Predictions

Three codes for representing auditory space were formulated in terms of single-neuron spatial tuning curves (Fig. 2B–D, top). In *the uniform place code*, all tuning curves were Gaussians with a standard deviation of 26°. For a set of 360 neurons, the tuning curves were centered at 1° intervals distributed evenly across the horizontal plane. In *the non-uniform place code*, 360 tuning curves were also centered at 1° intervals but their standard deviation varied. At 0°, the standard deviation was 15°, and at 180° it was 53°. For the intermediate locations the standard deviation changed linearly. In *the population rate code*, the tuning curves were Gaussians with a constant standard deviation of 64°. The curves were centered at 1° intervals at lateral locations from 80° to 100° and from 260° to 280°.

The predicted N1m response amplitude *R*_N1_ to each sound source location was determined as the sum of the activity levels of the neurons (Fig. 2B–D, bottom). The response amplitude to the probe when no adaptors were presented was *R*_1_ = Σ*_i_r_i_*(*p*), where *r_i_*(*p*) was the height of the tuning curve of neuron *i* at the location of the probe *p*. The presentation of an intervening adaptor was assumed to lead to a 50% decrease in the response amplitude of each neuron. This decrease was calculated relative to the response to the adaptor. Thus, the response amplitude in conditions where the adaptors were presented was *R*_2_ = Σ*_i_*[*r_i_*(*p*) −0.5*r_i_(*a*)], where *r_i_*(*a*) was the height of the tuning curve of neuron *i* at the location of the adaptor *a*. No negative activities were allowed. Thus, if *r_i_*(*p*) −0.5*r_i_(*a*) was negative it was set to zero before summing to the population response. Finally, the response amplitudes were expressed proportional to the amplitude of the unadapted response: *R*_N1_ = *R*_2_/*R*_1_.

According to single-unit recordings [37]–[39] and human neuroimaging data [44]–[47], the left- and right-preferring populations are not of equal size but, rather, the contralaterally tuned population is larger than the ipsilaterally tuned one. In the population rate code, the left- and right-tuned populations contribute with opposite patterns of the adaptation effect, each being more attenuated by adaptors in their preferred location. Thus, the relative sizes of the two populations could potentially have a significant impact on the compound activity represented by the N1m. The model prediction was, however, relatively insensitive to changes in the population sizes: The N1m responses to the probes in the left hemifield arose mainly from neurons tuned to the left hemifield while the contribution of those tuned to the right was very small (Fig. 6). Consequently, the effect of the neurons tuned to the right hemifield remained weak when their number was below 70% of all neurons. Thus, the predictions presented here based on a model where the populations are of equal size are similar to those obtained with other settings where at least 30% of neurons are tuned to the left hemifield. Some previous findings suggest that the difference between the relative sizes of the two populations may be more extreme than this, with especially the left hemisphere receiving predominantly contralateral input [46]. In terms of the current model, this would be reflected as the right hemisphere having a pattern of location-specific adaptation consistent with the prediction presented here and the left hemisphere showing an opposite pattern. In contrast, similar patterns obtained from the two hemispheres arise when they both contain a large proportion (>30%) of neurons tuned to the left hemifield.
